# Beyond the Abdominal Wall: Appendiceal Abscess Concealed Within a Spigelian Hernia

**DOI:** 10.3390/reports9020150

**Published:** 2026-05-13

**Authors:** Ioannis Katsarelas, Alexandra Panagiotou, Mohammad Husamieh, Ismini Kountouri, Periklis Dimasis

**Affiliations:** Department of General Surgery, General Hospital of Katerini, 60132 Katerini, Greece; alexandrapanayiotou.larissa98@gmail.com (A.P.); 3bbadi1997@gmail.com (M.H.); i.kountouri531@gmail.com (I.K.); dimasis@yahoo.com (P.D.)

**Keywords:** general surgery, emergency surgery, hernia, spigelian hernia, appendiceal abscess, computed tomography, interesting images

## Abstract

Spigelian hernias represent infrequent abdominal wall defects, and the incarceration of an inflamed appendix within the hernia sac is an even rarer clinical finding. We report an uncommon case in which a long-standing herniated appendix eventually progressed to perforation and abscess formation. Our report aims to illustrate this clinical transition to an atypical surgical emergency, while emphasizing that early Computed Tomography (CT) is vital for resolving diagnostic uncertainty and avoiding potential pitfalls in complex abdominal wall pathologies. Following prompt surgical intervention and hernia repair, the patient had an uneventful recovery and remained asymptomatic at the six-month follow-up.

Spigelian hernias constitute a distinct clinical entity, arising from defects in the Spigelian fascia—the aponeurotic layer between the rectus abdominis and the semilunar line [1]. These defects are predominantly localized within the “Spigelian hernia belt,” a transverse 6 cm wide zone above the interspinal plane [2,3]. While omental or small bowel herniation is typical, the presence of the appendix within the sac is highly unusual, and its progression to suppurative inflammation and perforation remains even more rare. Such cases are difficult to diagnose because of their location between the muscle layers. This report describes a rare clinical case of an elderly patient with an appendiceal abscess developing inside a Spigelian hernia, highlighting how important clinical suspicion and detailed CT imaging are for a timely diagnosis and accurate surgical management [4,5].

An 83-year-old male presented with a non-reducible mass in the right lower quadrant, located lateral to the right rectus sheath and superior to the pubic symphysis. The patient reported a 6-month history of a partially reducible bulge with intermittent dull ache, which had acutely progressed to sharp, localized pain and pyrexia (38.0 °C) within 24 h prior to admission. His medical history was significant for hypertension, type 2 diabetes mellitus, and hypercholesterolemia, while he was also receiving aspirin daily as antiplatelet medication. No prior abdominal surgery was reported, and no surgical scars were evident. The patient’s body-mass index (BMI) was within the normal range (23.7). Physical examination revealed a tender mass with localized guarding and slight overlying erythema, with no signs of generalized peritonitis (Figure 1).

Clinical assessment for a cough impulse and Valsalva maneuver was negative, confirming the non-reducible nature of the mass and raising suspicion for incarceration. Laboratory evaluation revealed elevated WBC count (21.35 × 10^3^/μL), alongside an elevated CRP level (18.16 mg/dL). Other biochemical markers were all within normal limits. Differential diagnosis included hernia incarceration, abscess, skin infection, appendicitis, diverticulitis and abdominal wall inflamed cyst. The patient was urgently admitted to the surgical department, and empirical intravenous broad-spectrum antibiotic therapy was initiated with piperacillin/tazobactam, alongside aggressive fluid resuscitation. Due to medical history being consistent for a hernia and clinical examination raising suspicion for inflammation, a contrast-enhanced CT scan was performed (Figure 2).

Based on the imaging findings being consistent with incarceration of the appendix in the Spigelian hernia sac, causing abscess formation, the patient underwent urgent surgical exploration. Given the diagnostic complexity, a trans-abdominal laparoscopic approach was initially adopted. Exploratory laparoscopy confirmed the Spigelian hernia, containing the appendix and excluded generalized peritonitis (Figure 3).

Primary management involved drainage of the purulent collection, followed by an attempt to mobilize the cecum and reduce the herniated appendix back to the peritoneal cavity. Due to firm inflammatory adhesions, a conversion to open exploration was required to ensure safe dissection. An oblique incision over the bulge was utilized. Following the division of subcutaneous fat tissue, the aponeurosis of the external oblique was incised to access the abscess cavity and the hernia sac, protruding through the Spigelian fascia (Figure 4).

Following complete mobilization from the internal oblique and transversus abdominis muscles, the hernia sac was opened. After obtaining intraoperative swabs for microbiological culture from the abscess cavity, a formal appendectomy was performed, and the surgical field was thoroughly irrigated and debrided. This was followed by primary repair of the fascial defect using non-absorbable interrupted sutures (Polypropylene #1). Mesh was intentionally avoided due to the relatively small size of the defect (2 cm) and the dirty-infected surgical field (CDC Class IV wound). Histopathological examination confirmed acute suppurative appendicitis with gangrenous areas and perforation, the presence of fecaliths, and transmural inflammatory infiltration extending to the mesoappendix, without evidence of malignancy.

Initial postoperative recovery was uneventful. The patient was maintained on intravenous piperacillin/tazobactam, as intraoperative cultures isolated Escherichia coli sensitive to the initial regimen. He was discharged on postoperative day 4 with his antibiotic therapy de-escalated to oral amoxicillin/clavulanic acid. A superficial surgical site infection (CDC Class I) occurred post-discharge, which was managed with bedside wound irrigation and completion of the oral antibiotic course, resulting in complete healing. Long-term follow-up at one year, including clinical examination and ultrasound, confirmed no evidence of hernia recurrence. The patient’s clinical course is summarized in the timeline below (Figure 5).

The clinical course of this patient, where a stable six-month history was followed by a rapid deterioration within just 24 h, highlights how unpredictable Spigelian hernias can be. This acute progression of a chronic condition, leading to an appendiceal abscess, is a rare event in clinical practice.

Herniated content of a Spigelian hernia is usually the greater omentum, while herniation of small intestine, colon, ovaries, Meckel’s diverticulum and the appendix have also been reported [6,7]. Spigelian hernias can have an acute presentation and often require urgent surgical intervention or cause chronic symptoms and an incidental discovery [8]. Our case combines many uncommon characteristics, with an acute-on-chronic presentation, herniated inflamed appendix and eventual abscess formation. Consistent with two independent reviews, our literature search identified only 18 reported cases of an appendix in a Spigelian hernia, making this the 19th documented instance [9,10]. Clinical presentations of these cases included both acute and chronic manifestations, with intraoperative findings of the appendix ranging from unremarkable, to perforation and abscess formation [9,10,11,12,13,14].

The literature data indicates a preference for open surgery over laparoscopic approaches, while conversion from laparoscopy to an open approach frequently occurs in cases of abscess formation, as was observed in our patient [9,10,14]. Specifically, the literature reports open surgery in 57% (11/19) of cases and laparoscopic intra-abdominal approaches in 26% (5/19). Consistent with our intraoperative findings, the literature suggests that the presence of localized sepsis and firm inflammatory adhesions may represent a relative contraindication to a pure laparoscopic approach, as two similar cases with abscess formation required conversion from a laparoscopic to an open approach. While we managed this emergency with a combined open/laparoscopic approach, the potentially emerging role of robotic surgery should be mentioned. In stable patients, robotic platforms may provide superior anatomical visualization and precision for complex repairs, especially in centers of adequate experience.

Regarding repair techniques, only 21% (4/19) of reported studies utilized mesh at the primary operation, with 16% (3/19) describing a normal appendix within the sac [10]. Current surgical consensus for incarcerated Spigelian hernias indicates that when sepsis is present, primary suture repair is superior to mesh-based techniques due to the risk of biofilm formation and mesh extrusion. In line with our clinical management, for small hernia defects (<3 cm), primary closure is the treatment of choice, adhering to the WSES guidelines for infected wounds and the Losanoff–Basson classification, which recommends against prosthetic materials in the setting of acute appendicitis within a hernia sac [15,16]. In cases of larger fascial defects where primary closure is not feasible, the use of biological or biosynthetic meshes may be considered to provide structural support while resisting infection. Alternatively, a staged approach involving open wound management followed by delayed anatomical repair remains a valid strategy for highly contaminated surgical fields.

In conclusion, the incarceration and perforation of the appendix within a Spigelian hernia represents a complex diagnostic and surgical challenge. The clinical significance of this report lies in the rare localization of an appendiceal abscess within the Spigelian fascia. The presence of a palpable abdominal wall mass should always raise clinical suspicion for an underlying hernia, even when inflammatory signs suggest superficial pathology. Surgeons must remain vigilant in managing atypical abdominal wall pathologies, ensuring the best outcome for the surgical patient.

## Figures and Tables

**Figure 1:**
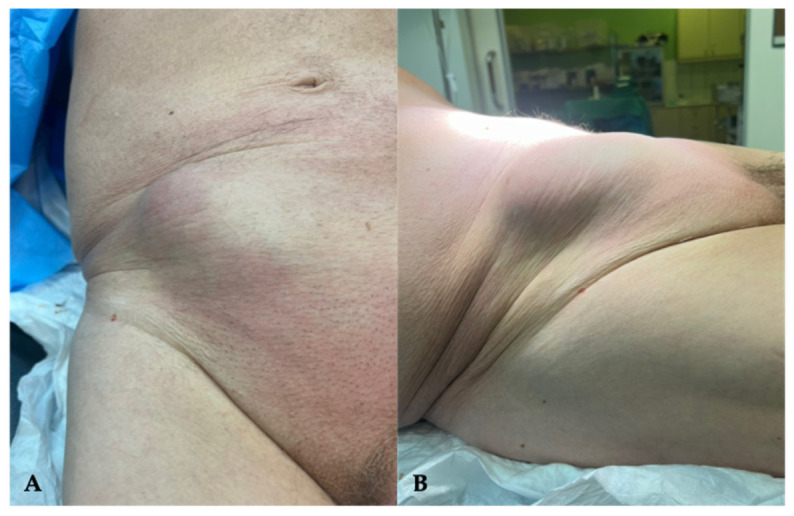
(**A**) Superior and (**B**) lateral clinical view of an 83–year–old male patient who presented with a non-reducible mass, located lateral to the right rectus sheath, superior to the pubic symphysis.

**Figure 2:**
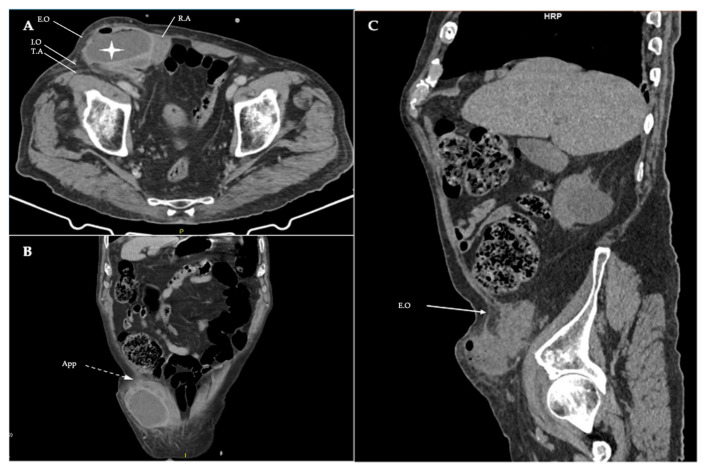
(**A**) Axial view of contrast-enhanced CT showing the organized abscess of the abdominal wall (white star) and the fascia defect of the Spigelian hernia, as well as the rectus abdominis (R.A), the right-side external oblique aponeurosis (intact—E.O), the internal oblique (I.O) and transversus abdominis (T.A). (**B**) Coronal view, highlighting the appendix (dashed arrow—App) protruding through the fascia defect. (**C**) Sagittal view, in which the muscle layers of the abdominal wall and the hernia orifice are best identified, with external oblique aponeurosis remaining intact (solid arrow—E.O).

**Figure 3:**
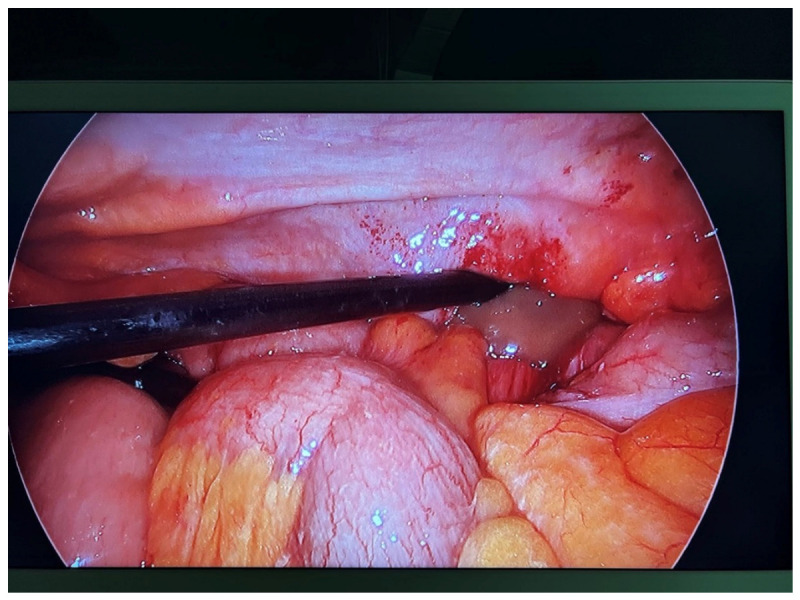
Intraoperative view of the exploratory laparoscopy.

**Figure 4:**
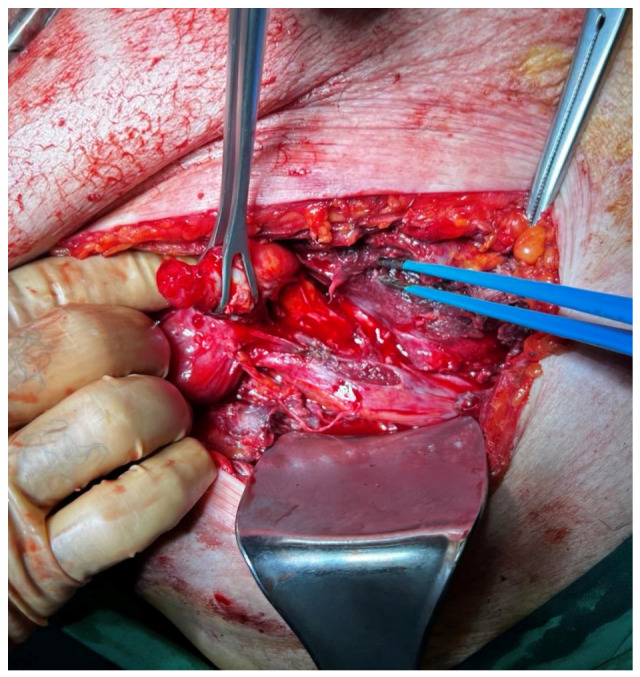
Open surgical view highlighting the perforated appendix with extensive wall thickening, retracted with Babcock forceps.

**Figure 5:**
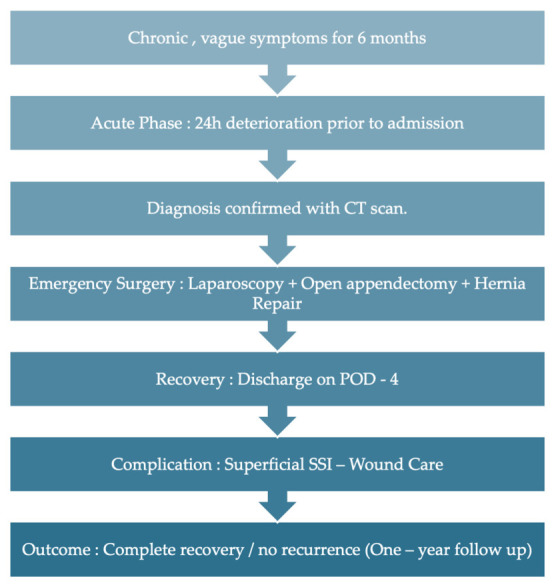
Timeline of the patient’s clinical course and management.

## Data Availability

The original contributions presented in this study are included in the article. Further inquiries can be directed to the corresponding author.
